# The Effects of Cucumber Mosaic Virus and Its 2a and 2b Proteins on Interactions of Tomato Plants with the Aphid Vectors *Myzus persicae* and *Macrosiphum euphorbiae*

**DOI:** 10.3390/v14081703

**Published:** 2022-08-01

**Authors:** Warren Arinaitwe, Alex Guyon, Trisna D. Tungadi, Nik J. Cunniffe, Sun-Ju Rhee, Amjad Khalaf, Netsai M. Mhlanga, Adrienne E. Pate, Alex M. Murphy, John P. Carr

**Affiliations:** 1 Department of Plant Sciences, University of Cambridge, Downing Street, Cambridge CB2 3EA, UK; w.arinaitwe@cgiar.org (W.A.); alex.guyon@slcu.cam.ac.uk (A.G.); trisnat@gmail.com (T.D.T.); njc1001@cam.ac.uk (N.J.C.); sjrhee825@gmail.com (S.-J.R.); ak37@sanger.ac.uk (A.K.); netsai.mhlanga@niab.com (N.M.M.); ap411@cam.ac.uk (A.E.P.); amm1013@cam.ac.uk (A.M.M.); 2 Alliance of Bioversity International and International Center for Tropical Agriculture (CIAT), Dong Dok, Ban Nongviengkham, Vientiane CB10 1RQ, Laos; 3 Sainsbury Laboratory, Cambridge University, Bateman St, Cambridge CB2 1LR, UK; 4 School of Life Sciences, Keele University, Newcastle ST5 5BG, UK; 5 Wellcome Sanger Institute, Wellcome Trust Genome Campus, Hinxton, Saffron Walden CB10 1RQ, UK; 6 National Institute for Agricultural Botany-East Malling (NIAB-EMR), West Malling ME19 6BJ, UK

**Keywords:** epidemiology, *NahG*, hemipteran vector, gas chromatography-mass spectrometry, viral suppressor of RNA silencing, RNA-dependent RNA polymerase

## Abstract

Cucumber mosaic virus (CMV), a major tomato pathogen, is aphid-vectored in the non-persistent manner. We investigated if CMV-induced volatile organic compounds (VOCs) or other virus-induced cues alter aphid–tomato interactions. Y-tube olfactometry showed that VOCs emitted by plants infected with CMV (strain Fny) attracted generalist (*Myzus persicae*) and Solanaceae specialist (*Macrosiphum euphorbiae*) aphids. *Myzus persicae* preferred settling on infected plants (3 days post-inoculation: dpi) at 1h post-release, but at 9 and 21 dpi, aphids preferentially settled on mock-inoculated plants. *Macrosiphum euphorbiae* showed no strong preference for mock-inoculated versus infected plants at 3 dpi but settled preferentially on mock-inoculated plants at 9 and 21 dpi. In darkness aphids showed no settling or migration bias towards either mock-inoculated or infected plants. However, tomato VOC blends differed in light and darkness, suggesting aphids respond to a complex mix of olfactory, visual, and other cues influenced by infection. The LS-CMV strain induced no changes in aphid–plant interactions. Experiments using inter-strain recombinant and pseudorecombinant viruses showed that the Fny-CMV 2a and 2b proteins modified tomato interactions with *Macrosiphum euphorbiae* and *Myzus persicae*, respectively. The defence signal salicylic acid prevents excessive CMV-induced damage to tomato plants but is not involved in CMV-induced changes in aphid–plant interactions.

## 1. Introduction

Cucumber mosaic virus (CMV) is a single-stranded, tripartite, positive-sense RNA virus, which encodes five proteins [1,2]. RNA 1 encodes a single translation product, the 1a protein, which has capping and helicase activities and forms part of the replicase complex. RNA 2 is the translation template for the 2a viral RNA-dependent RNA polymerase, and encodes the multifunctional 2b protein that is, among other things, a viral suppressor of RNA silencing. The 2b protein, which has multiple functions, and which localizes in the nucleus and cytoplasm of the host cell, is translated from the 4A sub-genomic RNA [3,4]. RNA 3 is the translational template for the CMV movement protein and encodes the coat protein, which is translated from sub-genomic RNA 4. The coat protein is required for assembly of virions and is the sole determinant for transmission by aphids [5,6]. The movement and coat proteins are required for cell-to-cell virus movement via the plasmodesmata and long-distance virus movement via the phloem [7,8,9].

CMV infects 1071 species in 521 genera from 100 families of monocots and dicots, including solanaceous hosts, such as tomato (*Solanum lycopersicum*) [10]. At least 60 aphid species vector CMV, and among the most important of these for transmission of the virus to solanaceous hosts are the cosmopolitan aphid *Myzus persicae* and the aphid *Macrosiphum euphorbiae* which has a narrower host range and is more specialised towards solanaceous plants [11,12,13]. Aphids vector CMV in the non-persistent manner, i.e., the virus does not circulate in the insect body, and there is thought to be a loose interaction between the viral coat protein and aphid proteins located in the acrostyle region of an aphid’s stylet [14,15,16]. Non-persistent transmission of CMV to uninfected plants and acquisition of the virus from infected hosts is favoured by brief probes of plant epidermal cells, and virus particles are loosely retained in the insect stylet for at most 2–4 h post-acquisition [6,17].

Viruses induce physiological and biochemical changes in susceptible host plants (virally modified plant phenotypes) that influence arthropod vector behaviour in ways that may enhance transmission [18,19]. Virally modified plant phenotypes that may influence aphid interactions with plants include leaf chlorosis, changes in leaf texture, accumulation of anti-feeding compounds, changes in the emission of volatile organic compounds (VOCs), changes in the levels of phytohormones or nutrients, and consequent effects on performance (growth, reproduction) of aphids on infected plants [20,21,22,23,24,25,26,27,28,29,30,31].

For CMV, the nature of virally modified plant phenotypes can vary with virus strain, plant species, and aphid species. For instance, the Fny strain of CMV (Fny-CMV, a Sub-Group IA strain) induces feeding deterrence against *Myzus persicae* in squash (*Cucurbita pepo*) and in *Arabidopsis thaliana* (ecotype Col-0) [24,32]. However, the LS-CMV strain (a Sub-Group II strain) induces no effects on aphid–plant interactions in *A. thaliana* [24], but both it and Fny-CMV induce increased susceptibility to *Myzus persicae* in tobacco (*Nicotiana tabacum*) [33]. *Aphis gossypii* and *Myzus persicae* were attracted by CMV-induced VOCs emitted by infected squash and cucumber (*Cucumis sativus*) [32,34], but although the VOC blend emitted by tobacco plants was altered by infection with Fny-CMV, this had no influence on the attractiveness of the plants to *Myzus persicae* [35]. Virally modified plant phenotypes may have dissimilar effects on different aphid species. Thus, when aphids of the polyphagous species *Myzus persicae* were confined on *A. thaliana* plants infected with Fny-CMV they were deterred from feeding from the phloem and their growth and reproduction were inhibited [24]. However, when two crucifer specialists were confined on *A. thaliana* plants infected with Fny-CMV, aphids of *Brevicoryne brassicae* grew normally but showed decreased fecundity, but aphids of *Lipaphis erysimi* were unaffected [36]. In contrast, the feeding and plant location behaviours of *Myzus persicae* and *Aphis fabae* (a legume specialist) were in broad terms affected similarly on CMV-infected common bean (*Phaseolus vulgaris*) plants, although *Myzus persicae* exhibited increased feeding difficulties on CMV-infected plants [28,29].

At least three CMV gene products influence CMV-induced alterations in host–aphid interactions. The 2b protein influences VOC emission in tobacco plants and in this host, it inhibits induction of resistance to *Myzus persicae* triggered by the 1a protein [33]. However, in *A. thaliana* the 2b protein has the potential to induce a strong form of resistance against *Myzus persicae* that is moderated though a direct interaction of the 2b protein with the CMV 1a protein [37]. Meanwhile, the CMV 2a protein induces feeding deterrence [24,38], which impels viruliferous aphids to emigrate from infected plants. In tomato and common bean, viruses induce production of VOC blends that attract bumblebees (*Bombus terrestris*) [39,40]. In the case of tomato plants, the emission of the bumblebee-attracting VOC blend is caused by the effects of the CMV 2b protein on small RNA pathways [39].

At this study’s inception, it was not known if CMV could modify interactions between tomato plants and aphids. We investigated if CMV infection, the 2b protein or other CMV gene products influenced interactions of aphids with tomato plants. We also investigated if changes in VOC emission or other virus-inducible, insect-perceivable cues influenced aphid behaviour. In part, these experiments were carried out to determine if any effects of tomato VOCs on aphid behaviour differed from, or were similar to, those previously shown to influence bumblebee behaviour. Additionally, given that virally modified plant phenotypes that affect plant–aphid interactions can have differential effects on non-specialist versus specialist aphids, we investigated the behaviours of *Myzus persicae* and of *Macrosiphum euphorbiae*, a solanaceous plant specialist, when these aphids were confined on CMV-infected tomato plants.

## 2. Materials and Methods

### 2.1. Aphids and Plants

Colonies of *Myzus persicae* (Sulzer) clone US1L [41] were maintained parthenogenetically on Chinese cabbage (*Brassica rapa* subspecies *pekinensis* (Lour) Hanelt) cv. Green Rocket F1 (Kings Seeds, Essex, UK). Colonies of a clone of *Macrosiphum euphorbiae* (Thomas) [42] were propagated parthenogenetically on plants of potato (*Solanum tuberosum* L.) cv. Desiree (Berrycroft Stores Ltd., Cambridge, UK). To contain aphids, plants and pots were wrapped in micro-perforated plastic bags (Seal Packaging, Bedfordshire, UK) and placed in an insect-proof fabric cage (Insect Cage Net, Carmarthen, Dyfed, UK). Aphid clones were generously provided by Rothamsted Research, Harpenden, UK.

Seeds for tomato (*Solanum lycopersicum* L.) cv. Moneymaker were obtained from Kings Seeds, UK and seeds for the *NahG*-transgenic tomato line SLJ7321 in the Moneymaker background [43] were generously provided by Prof. Jonathan Jones. *Nicotiana benthamiana* Domin. and *rdr6i*-transgenic *N. benthamiana* were used for virus propagation. Seeds of tomato and *N. benthamiana* were germinated on sterile moist filter paper in Petri dishes at 28 °C for 5 days before transfer of germinated seedlings to Levington M3 compost (Scotts, Chillingworth, Ipswich, UK). Plants were grown in a controlled environment room (Conviron, Manitoba, Canada) with a 16 h photoperiod under 200 µE·m^−2^·s^−1^ of photosynthetically active radiation at 22 °C and 60% relative humidity.

### 2.2. Virus Preparation, Plant Inoculation, and Virus Detection

Wild-type Fny-CMV and LS-CMV, or pseudorecombinant (reassortant) viruses were constituted by mixing in vitro-synthesised transcripts of infectious cDNA clones for the genomic RNAs of LS-CMV [44] and Fny-CMV [45] (generously provided by Prof. Peter Palukaitis). In some cases, transcripts for wild-type RNA 2 molecules were substituted with transcripts of infectious clones for a recombinant CMV RNA 2 encoding the Fny-CMV 2a protein and the LS-CMV 2b protein [46] (generously provided by Prof. Marilyn Roossinck), or for an RNA *2b* gene deletion mutant [24,33,38,47]. Plants of the wild-type *N. benthamiana* of the highly virus-susceptible accession [48] were used for propagation of all viruses except for Fny-CMVΔ2b [47], which was propagated in plants of the hypersusceptible transgenic *rdr6i*-*N. benthamiana* line [49]. Plants were mechanically inoculated on two lower leaves when 14 days old and virions isolated from systemically infected tissues 10–14 days later using the procedure described by Palukaitis [50].

Purified virions (4 µL of 500 ng/µL) were mechanically inoculated onto cotyledons of tomato plants at 10 days post-sowing, using Carborundum (Alfa Aesar, Heysham, UK). Control plants were mock inoculated with sterile water. CMV infection was confirmed in upper leaves using double antibody sandwich-enzyme linked immunosorbent assay (ELISA) kits sourced from Lynchwood Diagnostics (Grantham, UK). Absorbance readings at 405 nm were obtained using a Titertek Multiscan PLUS MKII (Huntsville, AL, USA) ELISA reader. Samples with absorbance values greater than twice the mean values of the negative controls (leaf samples of mock-inoculated plants) were considered positive [51]. For samples not fulfilling this criterion, infection by CMV was tested by RT-PCR using primers and conditions described by Rhee et al. [38].

### 2.3. Aphid Free-Choice Settling and Preference Assays and Olfactometry

Aphid free-choice settling assays [29,35] were performed with wingless *Myzus persicae* and *Macrosiphum euphorbiae*. Prior to use in experiments, aphid nymphs were starved at 4 °C for 24 h. Groups of 20 aphids were transferred into 1.5 mL microcentrifuge tubes. These tubes were placed equidistantly from plants that were 9 cm apart. Aphids that settled on either plant were counted at 1 and 24 h following aphid release. Experiments were carried out under normal illumination or covered to exclude visual cues. To determine how the progress of infection affected choices between mock-inoculated and CMV-infected plants, infected plants at 3, 9, and 21 dpi were used. Modified aphid free-choice assay was conducted with double-sided adhesive tape (Q-connect, Sheffield, UK) strips placed so that aphids approaching plants would be trapped, to indicate their initial direction of travel, which we refer to as a preference assay. Aphid choices were recorded at one hour following release. After optimisation, this assay was routinely carried out using tomato plants 9 days after inoculation with CMV or mock inoculation.

For Y-tube olfactometry individual starved aphids were released at the base of the Y-tube, downstream of the air stream and observed for at least 20 min. A choice for either air source was recorded when an aphid passed a marker at 2 cm up one of the olfactometer arms. Between experiments, the position of air sources was regularly swapped to control for any directional biases in the setup. Assays were carried out under uniform diffuse white light to eliminate visual cues. The apparatus was cleaned with acetone between experiments.

Choice and Y-tube olfactometry experiments with aphids were carried out at least three times. Statistical analyses on aphid choice tests were conducted using R v.4.0.2 [52], using binomial generalized linear mixed models, fitted using glmer in the lme4 package [53]. The experimental replicate was used as a random effect in fitting the models, allowing for systematic differences between replicates to be accounted for [40]. Wald tests assessing whether the fixed effect (i.e., the intercept) in fitted models differed significantly from 0 were used to determine whether the probability that aphids would settle on an infected plant at 1 and 24 h post-release significantly differed from 0.5. Confidence intervals (95% Wald intervals) on the fitted intercepts are shown in the figures. To account for multiple comparisons, *p*-values were subjected to the Holm–Bonferroni correction; in all cases this was done within the set of results corresponding to a single figure.

### 2.4. Volatile Organic Compound Entrainment and Analysis

VOC collection from virus-inoculated plants or mock-inoculated tomato plants at 9 dpi, in both light and dark conditions, was carried out by the dynamic headspace air entrainment method. VOCs were analysed by coupled gas chromatography-mass spectrometry (GC-MS) as described originally by Beale and colleagues [54], using the same equipment and analysis conditions recently described by Mhlanga et al. [40]. Data were analysed using Xcalibur software (Thermo Scientific, Loughborough, UK). Generated mass spectra were compared with known metabolites held in the National Institute of Standards and Technology spectral databases (http://www.nist.gov (accessed on 31 July 2022)) and by comparison with spectral peaks of authentic standards. Abundantly emitted individual VOCs were quantified using standard curves generated with standards for α-pinene, carene, *p*-cymene, (-)-*trans*-caryophyllene, and nonanal and analysed statistically by analysis of variance and Tukey’s *post hoc* test. VOCs for which standards were unavailable were quantified as α-pinene equivalents. Volatile entrainment was done over 24 h and repeated three times using fresh plants and cleaned equipment between replicates. All glass and metallic parts used were washed and heat sterilised. Tomato plants were contained in glass bell jars clamped to two semi-circular metallic plates with a hole in the centre to accommodate the stem [39]. Dark conditions for plants were created by wrapping glass bell jars containing plants with aluminium foil during the sampling period.

## 3. Results

### 3.1. CMV Alters the Settling Behaviour of Aphids on Tomato Plants

**Figure 1:**
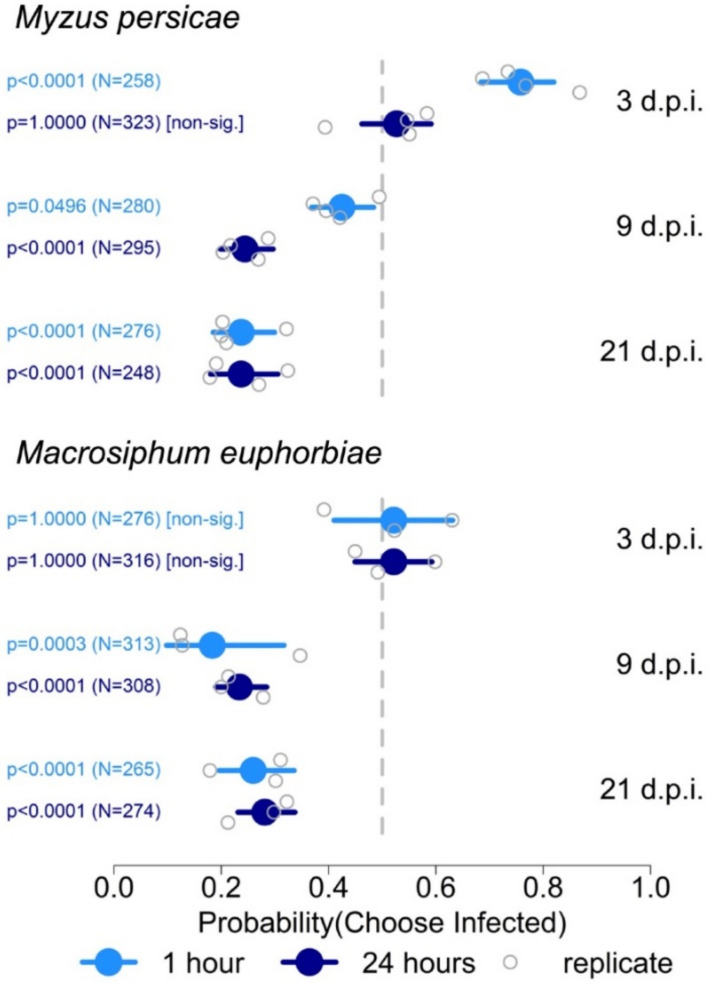
Summary of free-choice aphid preference assays to determine the likelihood of settling by aphids (*Myzus persicae* and *Macrosiphum euphorbiae*) on mock-inoculated tomato plants or plants infected with Fny-CMV. The *x*-axis shows the range of the 95% credible interval of the probability of settlement of aphids on infected plants at 1 and 24 h after aphid release, with values for each independent experiment (replicate: four for *Myzus persicae* and three for *Macrosiphum euphorbiae*) indicated. Free choice experiments were carried out using tomato plants at three time points following mock inoculation or inoculation with CMV (days post-inoculation: dpi). The total number of aphids (N) investigated for each comparison is indicated and statistical significance or non-significance was determined using binomial tests. The vertical grey dotted lines indicate a probability of 0.5 of settlement on infected versus mock-inoculated plants, i.e., no preference.

Tomato plants were inoculated on cotyledons with Fny-CMV, and systemic movement of the virus was detectable at 3 days post-inoculation (dpi) when it had accumulated to detectable levels in non-inoculated leaves. This was several days before onset of the classic symptoms of CMV infection in tomato (stunting, chlorosis, and leaf malformation [55]) (Figure S1). The settling preferences (at 1 and 24 h post-release) of *Myzus persicae* and *Macrosiphum euphorbiae* at 3, 9, and 21 dpi was investigated using aphid free-choice assays. Experiments were carried out four times independently (Spreadsheet S1). When plants were used early in infection (3 dpi), observations at 1 h following aphid release showed that *Myzus persicae* consistently displayed a significantly increased likelihood of settling on plants infected with Fny-CMV rather than on mock-inoculated plants (Figure 1). However, this preference for infected plants at 3 dpi was transient and by 24 h post-release the likelihood of *Myzus persicae* settling on infected or on mock-inoculated plants was similar (Figure 1). In a single experiment performed with *Macrosiphum euphorbiae*, aphids showed a preference for settling on infected plants at 3 dpi, but this did not occur consistently across all experiments (Figure 1; Spreadsheet S1). At later stages of infection, the likelihood of aphids of either species settling on mock-inoculated plants rather than on plants infected with Fny-CMV increased. This became increasingly consistent as infection progressed and by 21 dpi aphid settlement for both species occurred predominantly on non-infected plants at 1 and 24 h post-release (Figure 1; Spreadsheet S1). Data for mock-inoculated versus mock-inoculated choice tests are presented in Figure S2.

**Figure 2:**
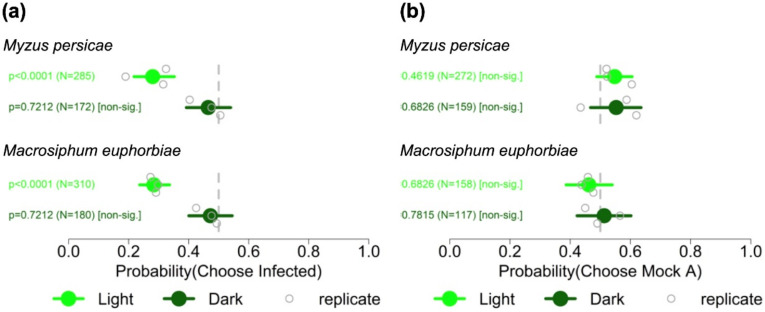
Summary of adhesive strip trapping experiments to determine the initial preferences of aphids for mock-inoculated tomato plants or plants infected with Fny-CMV in the dark or under illumination. Aphids (*Myzus persicae* and *Macrosiphum euphorbiae*) were released between pairs of tomato plants that had been mock-inoculated or inoculated with Fny-CMV (21 days previously) (**a**) or between pairs of mock-inoculated plants (**b**), to control for any additional stimuli or biases in the experimental set-up under illumination (Light) or in the dark. Adhesive traps were present between the release point and each plant, and at 1 h post-release trapped aphids were counted to assess their initial direction of travel. The *x*-axis shows the range of the 95% credible interval of the probabilities of travel of aphids towards the variously treated plants with values for each of three independent experiments (replicate) indicated. The total number of aphids (N) investigated for each comparison is indicated and statistical significance or non-significance was determined using binomial tests. The vertical grey dotted lines indicate a probability of 0.5 of a preference for migrating towards infected versus mock-inoculated plants, i.e., no significant difference in migration.

Aphid settling behaviour can potentially be influenced by visual, olfactory, or tactile cues, or various combinations thereof. To investigate if CMV-induced visual cues influence the aphid responses seen in free choice experiments (Figure 1), we adapted the free choice settling assays to identify the initial directions of migration of the aphids in the first hour after their release (Figure 2; Spreadsheet S1). This was done by including adhesive barriers to trap the aphids and carried out under illumination or in darkness to identify the preference of aphids to initially move towards one or other plant without the opportunity for the insects to experience plant taste or contact cues. Under light, aphids of both *Myzus persicae* and *Macrosiphum euphorbiae* were more likely to move towards mock-inoculated plants than towards plants infected with Fny-CMV at 9 dpi (Figure 2a). In contrast with the results obtained under light, in darkness there were no statistically significant differences in the likelihood of aphids of either species to move towards mock-inoculated or infected plants (Figure 2a). In the dark, fewer aphids appeared to migrate towards either mock-inoculated or infected plants but this was not investigated in detail.

The results show that as infection developed, CMV-infected tomato plants became less attractive to aphids of both species (Figure 1). The trapping experiments under normal illumination indicate that either olfactory or visual cues orientate the aphids towards mock-inoculated plants (Figure 2). While experiments in the dark suggest that visual cues are more important than olfactory cues, they may also indicate a reluctance of aphids to migrate at all in darkness, or that emission by plants of aphid-perceivable VOCs is altered qualitatively or quantitatively when they are placed in darkness.

### 3.2. Myzus persicae and Macrosiphum euphorbiae Preferred Odours Emitted by Plants Infected with Fny-CMV

**Figure 3:**
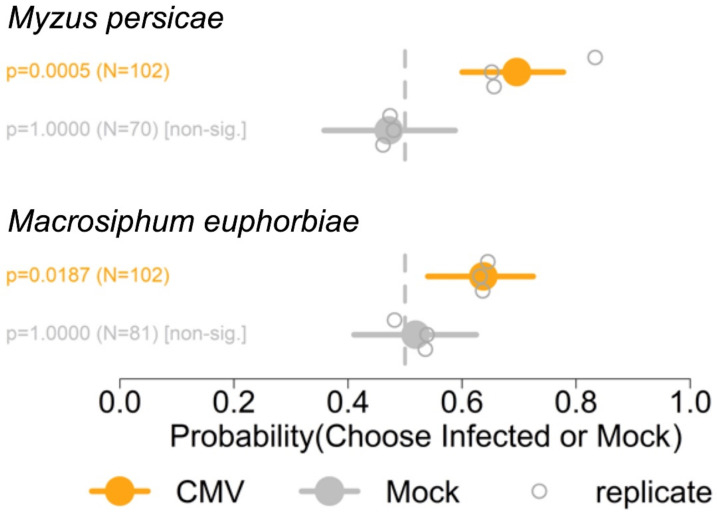
Summary of Y-tube olfactometry experiments to determine the preferences of aphids (*Myzus persicae* and *Macrosiphum euphorbiae*) for volatile organic compound blends emitted by mock-inoculated (Mock) tomato plants versus plants infected with cucumber mosaic virus (CMV, Fny starin) at 21 days post-inoculation. The *x*-axis shows the range of the 95% credible interval of the probabilities for preferences of aphids towards volatile organic compound blends emitted by infected or mock-inoculated plants. Three independent experiments (replicates) were carried out. The total number of aphids (N) observed for each comparison is indicated. The statistical significance or non-significance was determined using binomial tests. The vertical grey dotted lines indicate a probability of 0.5 of settlement on infected versus mock-inoculated plants, i.e., no preference for or against VOC blends of infected plants.

Y-tube olfactometry was used to examine whether virus-induced changes in the emission by plants of aphid-perceivable VOCs were involved in determining CMV-induced changes in aphid–tomato interactions. In this assay design, aphids cannot be influenced by visual or tactile cues from the plants. Aphids of both species, *Myzus persicae* and *Macrosiphum euphorbiae*, were more likely to move into the olfactometer arm conveying VOCs emitted by plants infected with Fny-CMV than into the arm delivering VOCs from mock-inoculated plants (Figure 3). Thus, aphids of both species exhibited an innate preference for odours emitted by CMV-infected over those emitted by mock-inoculated tomato plants. However, taking into account the results of free-choice (Figure 1) and trapping experiments (Figure 2), our work suggests that responses to VOCs cannot solely explain aphid settling behaviour.

### 3.3. The Effect of Illumination on CMV-Induced Changes in Plant VOC Emission

**Figure 4:**
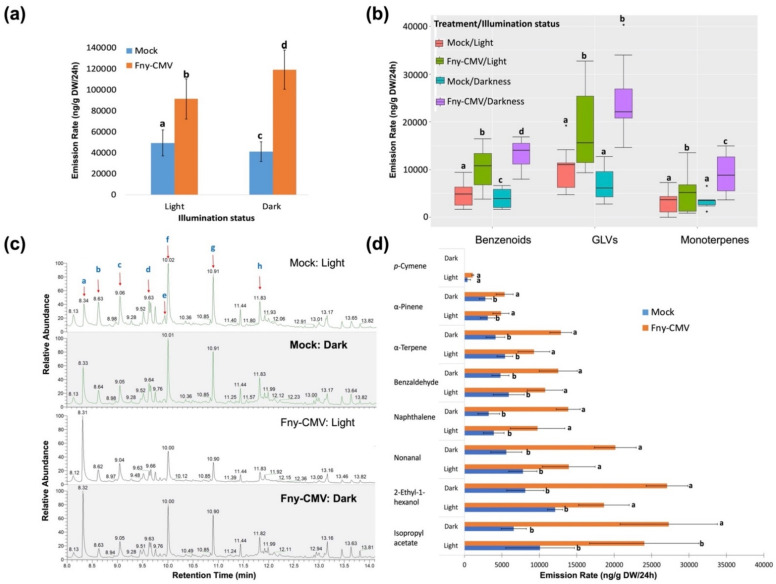
Analysis of volatile organic compounds (VOCs) emitted by infected and mock-inoculated tomato plants in darkness or under illumination. Gas chromatography and mass spectrometry were used to detect quantitative and qualitative differences in the VOCs emitted by mock-inoculated tomato plants or plants infected with Fny-CMV (at 21 days post-inoculation) and collected by dynamic headspace trapping. Quantitative differences in overall VOC emission (**a**) and for specific groups of VOCs: benzenoids, green leaf volatiles (GLVs), and monoterpenes (**b**). (**c**) Chromatograms of VOCs from tomato plants that had been mock-inoculated (upper two chromatograms) or infected with Fny-CMV (lower two chromatograms) under light and dark conditions. Blue lettering on the uppermost chromatogram indicates peaks for abundant VOCs emitted by mock-inoculated plants under light: a, isopropyl acetate; b, α-pinene; c, benzaldehyde; d, α-terpene; e, *p*-cymene; f, 2-ethyl-1-hexanol; g, nonanal; and h, naphthalene. (**d**) Quantitative differences in emission for specific VOCs emitted by infected versus mock-inoculated plants in light and dark conditions. Error bars in panels (**a**,**d**) indicate standard error around the mean. Different lowercase letters in panels (**a**–**c**) indicate statistically significant differences in emission rate using Tukey’s post hoc HSD test with ANOVA at α = 0.05.

We investigated if placing plants in darkness affects their emission of VOCs. If there are differences in VOC emission by mock-inoculated plants or plants systemically infected with Fny-CMV, this might provide an explanation for the contrasting results of the trapping experiments under illumination and darkness (Figure 2). Dynamic headspace collection showed plants infected with Fny-CMV emitted larger quantities of VOCs compared to mock-inoculated plants (*p* = 1.5 × 10^−9^, d = 1, F-value = 44.8: Figure 4a), and this was true when dynamic headspace volatile collection was carried out from unshaded plants or from plants in foil-covered bell jars. However, infected plants emitted significantly greater quantities of VOCs when covered than when uncovered (ANOVA with *post hoc* Tukey HSD test, *p* = 0.045). Mock-inoculated plants emitted similar quantities of VOCs whether or not they were covered during the headspace collection period (Figure 4a).

VOCs were disaggregated by chemical category. It was found that the emission rate of different VOC classes by infected plants varied significantly between light and dark conditions (ANOVA test: *p* = 2.81 × 10^−15^, df = 11, F value = 15.2) (Figure 4b). Several of the most abundant VOCs emitted by both virus-infected plants and mock-inoculated plants were green leaf volatiles (GLVs). These are C_6_ compounds that can act as semiochemical cues to aid herbivorous insects during host selection and their emission by plants is known to be affected by pathogen infection [56]. GLVs include: isopropylacetate; 2-ethyl-1-hexanol and nonanal; the benzenoids benzaldehyde and naphthalene; and the aromatic terpenes α-terpene, α-pinene, and *p*-cymene (Figure 4c,d). Both virus-infected and mock-inoculated plants produced greater quantities of GLVs, and to a lesser extent, benzenoid and monoterpenoid emissions were increased. No *p*-cymene was detected in blends emitted by tomato plants in the dark (Figure 4c,d). The results show that the VOC blends emitted by infected and non-infected tomato plants are quantitatively and qualitatively different when the plants are illuminated or placed in darkness.

### 3.4. CMV RNA 2 Influences Interactions between Tomato Plants and Aphids

**Figure 5:**
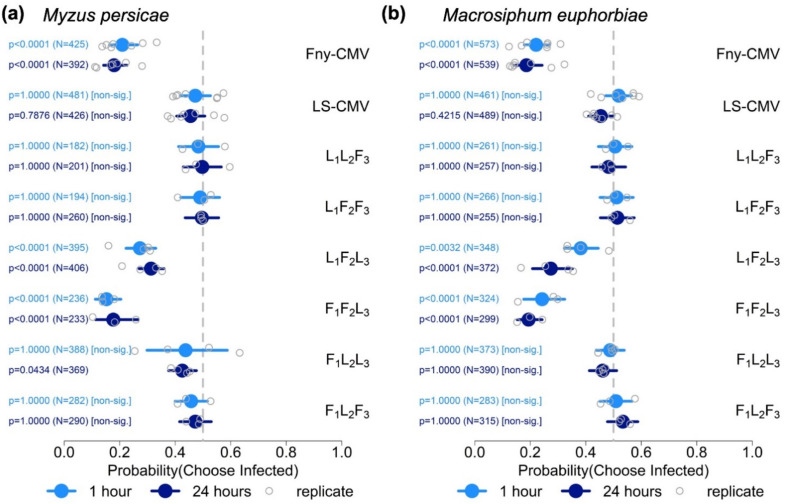
Summary of free-choice aphid preference assays for (**a**) *Myzus persicae* and (**b**) *Macrosiphum euphorbiae*) using mock-inoculated tomato plants or plants infected with wild-type LS-CMV, wild-type Fny-CMV or inter-strain pseudorecombinant (reassortant) viruses (at 21 days post-inoculation). The *x*-axis shows the range of the 95% credible interval of the probability of settlement of aphids on infected plants at 1 and 24 h after aphid release, with values for each independent experiment (replicate). The total number of aphids (N) investigated for each comparison is indicated and statistical significance or non-significance determined using binomial tests. The vertical grey dotted lines indicate a probability of 0.5 of settlement on infected versus mock-inoculated plants, i.e., no settlement preference.

Aphids of both species were less likely to settle on tomato plants infected by Fny-CMV than on mock-inoculated plants (Figure 1). However, preliminary experiments showed that infection with the LS strain of CMV (LS-CMV), a milder strain in tomato than Fny-CMV [57], did not result in any increase or decrease in settlement by either *Myzus persicae* or *Macrosiphum euphorbiae* (Figure S3). To attempt to identify the viral gene sequences conditioning the induction by Fny-CMV of resistance to aphid settlement, pseudorecombinant (reassortant) viruses were constructed by mixing in vitro-synthesized genomic RNA segments of Fny-CMV (indicated by ‘F’) with those of LS-CMV (denoted by ‘L’) (Figure 5). The results of free choice settling bioassays with these six pseudorecombinants are shown in Figure 5. Aphids of both species were more likely to settle on mock-inoculated plants than on plants infected with either L_1_F_2_L_3_ or F_1_F_2_L_3_ (Figure 5). The results with *Myzus persicae* were consistent across multiple experiments (Figure 5a) but less consistent with *Macrosiphum euphorbiae* (Figure 5a). Experiments with the pseudorecombinant virus F_1_L_2_L_3_ showed that it induced no effects in tomato that affected the probability of *Macrosiphum euphorbiae* settling on mock-inoculated versus infected plants (Figure 5b), but the results with *Myzus persicae* were less conclusive (Figure 5a). Results with L_1_F_2_L_3_ and F_1_F_2_L_3_ suggested that Fny-CMV RNA2, or one or both of the proteins it encodes, are responsible for conditioning changes in interactions between tomato plants and aphids. However, the results with L_1_F_2_F_3_ do not appear to be consistent with this idea, as aphids of neither species showed any preference for or against settling on plants infected with this pseudorecombinant virus (Figure 5). ELISA indicated that L_1_F_2_F_3_ accumulated poorly in tomato, which may explain why it did not induce any apparent effect on host–aphid interactions, although viral titre *per se* does not always have an effect on CMV-induced changes in vector–host interactions (for example, see reference [22]).

**Figure 6:**
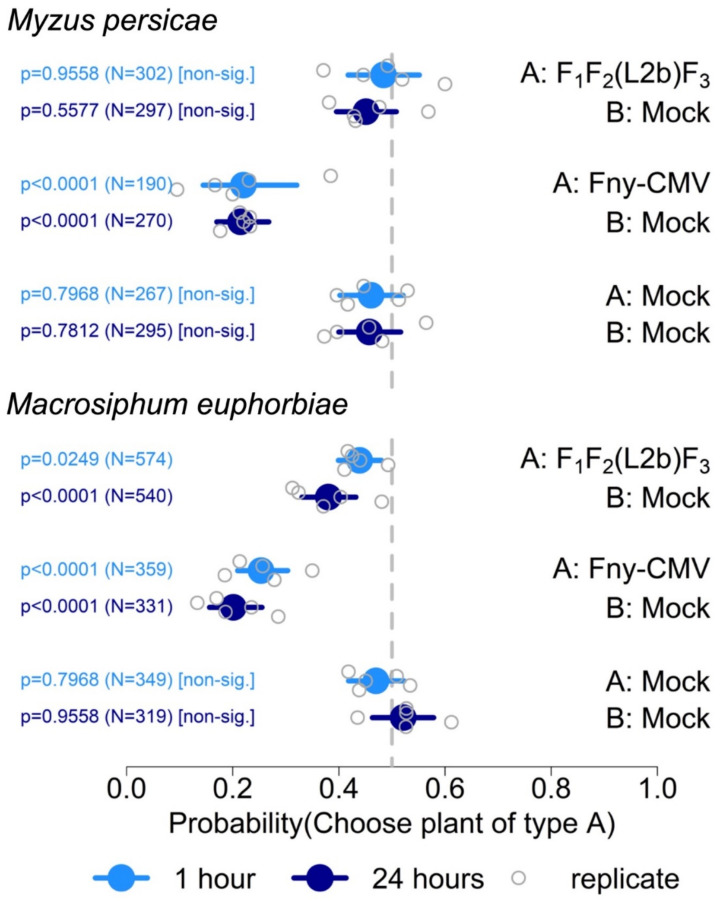
Summary of free-choice aphid preference assays using mock-inoculated tomato plants or plants infected with wild-type Fny-CMV or a recombinant CMV expressing the 2b protein of LS-CMV at 21 days post-inoculation. The *x*-axis shows the range of the 95% credible interval of the probability of settlement of aphids (*Myzus persicae* or *Macrosiphum euphorbiae*) on infected plants at 1 and 24 h after aphid release, with values for each independent experiment (replicate). The total number of aphids (N) investigated for each comparison is indicated and statistical significance or non-significance determined using binomial tests. The vertical grey dotted lines indicate a probability of 0.5 of settlement on infected versus mock-inoculated plants, i.e., no settlement preference.

To test the hypothesis that RNA 2 or its gene products, the 2a and 2b proteins, conferred Fny-CMV with the ability to modify tomato–aphid interactions, free-choice settling experiments were carried out using a recombinant CMV, F_1_F_2_(L2b)F_3_. This was produced by inoculating plants with a mixture of synthetic transcripts of the wild type genomic RNAs 1 and 3 of Fny-CMV with a recombinant RNA 2 in which the LS-CMV 2b open reading frame and 3′ untranslated region replaces that of Fny-CMV RNA2 (originally named F_2a_LS_2b_ [46]). Free-choice settling assays were done using plants at 9 dpi. In five independent experiments, aphids of *Myzus persicae* showed no significant differences in settling on tomato plants infected with F_1_F_2_(L2b)F_3_ versus mock-inoculated plants at either 1 or 24 h post-release (Figure 6). In assays using *Macrosiphum euphorbiae*, aphids showed no bias at 1 h post-release for settling on plants that had been mock-inoculated or infected with F_1_F_2_(L2b)F_3_ (Figure 6). However, by 24 h post-release, in four out of five experiments, significantly fewer aphids of *Macrosiphum euphorbiae* settled on plants infected with F_1_F_2_(L2b)F_3_ than on mock-inoculated plants (Figure 6).

**Figure 7:**
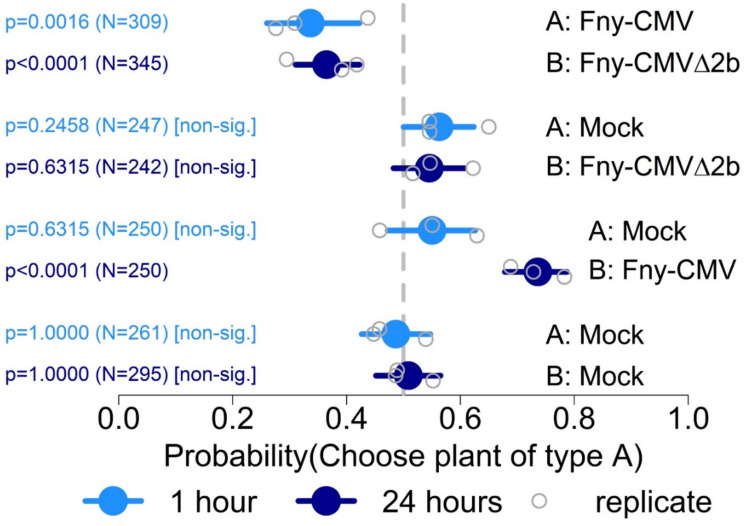
Summary of *Myzus persicae* settlement assays on tomato plants infected with Fny-CMVΔ2b versus plants infected with wild-type Fny-CMV or mock-inoculated plants at 21 days post-inoculation. Aphids were allowed to choose between settlement on two plants (plant A or B in various plant treatment combinations as indicated). The *x*-axis shows the range of the 95% credible interval of the probability of settlement of aphids (*Myzus persicae* or *Macrosiphum euphorbiae*) on infected plants at 1 and 24 h after aphid release, with values for each independent experiment (replicate). The total number of aphids (N) investigated for each comparison is indicated and statistical significance or non-significance was determined using binomial tests. The vertical grey dotted line indicates a probability of 0.5 of settlement on infected versus mock-inoculated plants, i.e., no preference.

The results obtained using the recombinant virus F_1_F_2_(L2b)F_3_ suggested that for *Myzus persicae*, but not for *Macrosiphum euphorbiae*, the Fny-CMV 2b protein is the most important determinant for the induction of resistance to aphid settlement on tomato. To confirm this, additional settling assays with *Myzus persicae* were carried out using tomato plants infected with the *2b* gene deletion mutant Fny-CMVΔ2b (Figure 7). In three independent experiments it was found that there were no significant differences in aphid settlement on plants infected with Fny-CMVΔ2b versus mock-inoculated plants. When aphids were allowed to choose between plants infected with Fny-CMVΔ2b and plants infected with wild-type Fny-CMV, a significantly larger proportion settled on plants infected with Fny-CMVΔ2b (Figure 7). Taken together, the results using pseudorecombinant viruses (Figure 5), as well as F_1_F_2_(L2b)F_3_ (Figure 6) and Fny-CMVΔ2b (Figure 7), show that in tomato Fny-CMV RNA 2 influences interactions with *Myzus persicae* and *Macrosiphum euphorbiae*, and that the Fny-CMV 2b protein is a major determinant of virally induced changes in aphid settling behaviour for *Myzus persicae* but not for *Macrosiphum euphorbiae*.

### 3.5. Investigating the Effect of Salicylic Acid on CMV-Induced Changes in Aphid–Tomato Interactions

The defence signal salicylic acid (SA) is an important factor in basal resistance to viruses, although its biosynthesis is increased by certain viruses, including CMV, in susceptible hosts [58,59,60]. To determine if SA-mediated signalling plays any role in CMV-induced changes in aphid–tomato interactions, we investigated aphid settling behaviour for *Myzus persicae* and *Macrosiphum euphorbiae* on *NahG*-transgenic tomato plants (Figure S4). During preliminary experiments it was noted that *NahG*-transgenic tomato plants are hypersusceptible to infection with Fny-CMV and by 10 dpi began to die (Figure S5), and therefore plants were used for aphid experiments at 7 dpi.

Decreasing SA accumulation in tomato did not influence CMV-induced changes in aphid settling preferences between mock-inoculated plants and plants infected with Fny-CMV (Figure S4; Supplementary Spreadsheet 1). This was the case for both *Myzus persicae* and *Macrosiphum euphorbiae*. In free-choice tests in which aphids were allowed to choose between settling on infected versus mock-inoculated plants, and regardless of whether one or both plants expressed the *NahG*-transgene, aphids preferred to settle on mock-inoculated plants (Figure S4). Experiments in which aphids chose between mock-inoculated *NahG*-transgenic and mock-inoculated non-transgenic plants showed that plants with diminished levels of SA are neither more nor less inherently attractive to either *Myzus persicae* or *Macrosiphum euphorbiae* (Figure S4). However, in two out of three experiments with *Myzus persicae* in which aphids were allowed to choose between infected *NahG*-transgenic plants and infected non-transgenic plants, by 24 h post-release aphids exhibited a preference for settling on *NahG*-transgenic plants that were infected with Fny-CMV (Figure S4).

## 4. Discussion

### 4.1. The Effects of Fny-CMV on Interactions of Tomato with Aphids

Fny-CMV induced changes in tomato plants that altered their susceptibility to infestation by the generalist aphid *Myzus persicae* and to *Macrosiphum euphorbiae*, an aphid that is more adapted to solanaceous hosts. The effects of Fny-CMV on host-aphid relations changed as infection of the plant progressed, and there were differences between the settling behaviour exhibited by aphids of the two species. At an early stage of systemic infection of tomato plants (i.e., at 3 dpi) *Myzus persicae* preferred to settle on infected plants rather than on mock-inoculated plants, but we infer that at later stages of infection hosts became increasingly repellent to *Myzus persicae*, since aphids of this species began to favour settling on mock-inoculated plants over plants infected with Fny-CMV. Using the epidemiological framework and terminology proposed by Donnelly et al. [18], the virally modified plant phenotype evoked by Fny-CMV in the interactions between infected tomato plants and *Myzus persicae* is ‘attract and deter’. That is, the virus induced a state that will initially attract vectors to an infected plants before impelling them to migrate away and carry viral inoculum to other hosts, which modelling indicates is likely to accelerate local plant-to-plant transmission [18]. The effects on *Macrosiphum euphorbiae* differed. Early in infection, there was less consistent evidence that infected tomato plants were more attractive to *Macrosiphum euphorbiae*; rather, there was no change in attractiveness to aphids, but as infection developed, these aphids, like those of *Myzus persicae*, appeared to be repelled by plants infected by Fny-CMV. Although not strictly a virally induced ‘attract and deter’ phenotype (as defined in [18]), we suspect that the effect is still likely to increase the probability of onward transmission since individuals of *Macrosiphum euphorbiae* that settle on infected plants will be later impelled to migrate and will likely carry inoculum with them.

### 4.2. Inter-Strain Differences in CMV-Induced Changes in Aphid–Tomato Interactions and the Role of CMV RNA 2

Infection by LS-CMV, which reaches a comparable viral titre to Fny-CMV in tomato [57], did not influence the settling choices of either *Myzus persicae* or *Macrosiphum euphorbiae*. The difference in the ability of these two strains to induce deterrence to aphid settling in tomato is reminiscent of what has been observed for Arabidopsis in which Fny-CMV, but not LS-CMV, induces resistance to *Myzus persicae* [24,38]. This difference between LS-CMV and Fny-CMV allowed us to use inter-strain pseudorecombinant viruses to identify the viral genomic RNA(s) responsible for induction of resistance to aphid infestation in tomato. These experiments indicated that RNA 2 of Fny-CMV played the strongest role in the induction of resistance to aphid settlement, which is consistent with what has been observed for Arabidopsis [24,38].

Perhaps due to differences in titre, some experiments with pseudorecombinant viruses were inconclusive. Therefore, experiments were also carried out with the *2b* gene deletion mutant Fny-CMVΔ2b and the recombinant virus F_1_F_2_(L2b)F_3_ to determine which of the two proteins encoded by CMV RNA 2 were critical in modifying aphid–tomato interactions. These experiments indicated that the Fny-CMV 2b protein is the predominant factor conditioning the repellence of infected tomato plants to settlement by *Myzus persicae* but not to *Macrosiphum euphorbiae*. Thus, the results do not rule out effects by other viral proteins or of viral RNA but point most strongly towards a role for the CMV 2a protein (the other protein encoded by RNA 2) in inducing cues that influence *Macrosiphum euphorbiae*, but not *Myzus persicae*. That different viral gene products influence interactions with each aphid suggests that, on tomato, *Macrosiphum euphorbiae* and *Myzus persicae* respond to different virus-induced cues.

These results contrast with findings using similar analyses of the effects of Fny-CMV infection on interactions of Arabidopsis and tobacco with *Myzus persicae*. With tobacco, the use of LS-CMV/Fny-CMV pseudorecombinants and Fny-CMVΔ2b showed that the respective 2b proteins of Fny-CMV and LS-CMV both counteract elicitation of a very strong resistance against *M. persicae* by the 1a protein of Fny-CMV [33]. Contrastingly, in infected Arabidopsis, it is the Fny-CMV 2b protein that induces a strong resistance to aphids, and it is the 1a protein which counteracts this; the consequence being that a 2a protein-induced feeding deterrence predominates during infection [24,37,38]. The results of our current study indicate that in tomato the Fny-CMV 2b protein induces a form of resistance to *Myzus persicae* that is likely to favour onward transmission of the virus, i.e., the role that is fulfilled in Arabidopsis by the 2a protein. It is unknown at this point whether the effects of the Fny-CMV 2b protein are modulated by the CMV 1a protein as they are in Arabidopsis [37] or if the 2b protein exerts its effects on tomato–aphid interactions through its ability to bind to ARGONAUTE 1 [61], or to small RNAs [62,63], or to other potential interactors such as JAZ proteins [64]. Nonetheless, what is becoming increasingly clear is that whilst three CMV gene products, the 1a, 2a, and 2b proteins, appear to determine changes in host-aphid interactions, the precise effects of these three CMV proteins differ markedly between plant host species and aphid vector species, which are matters needing additional investigation. Interestingly, a recent paper reported that a satellite RNA of CMV gave rise to small RNAs that were ingested by aphids, increasing the birth rate of winged morphs [65]. It will be interesting to see in future work how the indirect effects of CMV on aphids (i.e., virally induced plant phenotypes), and direct effects on vectors mediated by through satellite RNA-derived small RNAs, combine to influence vector behaviour on different hosts and with different aphid species.

### 4.3. To What Cues Are Aphids Responding?

The Fny-CMV 2b protein has been shown to influence the emission of bumblebee-perceivable VOCs by Arabidopsis and tomato plants [39]. However, aphids are not only influenced by VOCs, but also by several other cues, including visual stimuli. For example, although the Fny-CMV 2b protein induced changes in the VOC blend emitted by tobacco plants, these changes did not influence the plants’ attractiveness to *Myzus persicae* [35]. Virus-induced changes in visual stimuli influencing host-vector interactions are well documented. Ajayi and Dewar [66] found that colour changes induced by barley yellow dwarf virus in barley and oat plants made infected plants more attractive and susceptible to settlement by *Sitobion avenae* and *Metopolophium dirhodum*. Flight chamber experiments showed that *Acyrthosiphon pisum* was more attracted to tic bean plants infected by bean yellow mosaic, which display chlorosis, than to healthy plants [21].

Our Y-tube olfactometry showed that in the absence of any other cues *Myzus persicae* and *Macrosiphum euphorbiae* were more attracted to VOCs emitted by tomato plants infected with Fny-CMV than to the VOC blend of mock-inoculated plants. To this extent the effects of VOCs of virus-infected plants on aphid behaviour were similar to those observed for bumblebees, which are attracted by the VOCs emitted by CMV-infected tomato and bean plants [39,45]. However, when free-choice assays were modified, using adhesive strips, to determine the initial preference of aphids to move towards infected or mock-inoculated plants, most aphids were found to have moved in the direction of the mock-inoculated plants. When preference assays were carried out in darkness, fewer aphids migrated away from their point of release. The data suggest that *Myzus persicae* and *Macrosiphum euphorbiae* are influenced by virus-infected plants through a complex mix of visual and olfactory cues. The Y-tube olfactometry results suggest that virus-induced VOCs are more important at a distance when other cues are unavailable. However, our GC-MS results show that the VOC blends emitted by mock-inoculated and CMV-infected plants change when plants are placed in darkness, and we must conclude that some of our observations of light/dark differences in aphid behaviour may have been confounded by light-modulated effects on plant biochemistry.

### 4.4. The Relationship of CMV-Induced Salicylic Acid Accumulation to Aphid Infestation

CMV infection induces SA-regulated genes in tobacco, and it was inferred by Shi et al. [67] that SA mediates the inhibition of *Myzus persicae* fecundity and survival on tobacco (cv. Samsun NN) by CMV (probably the AN strain [68]). The CMV 2b protein, which in this study we found to be important in modifying tomato–aphid interactions, is known to prime SA biosynthesis and to subvert SA-induced virus resistance [58,59,69]; further suggesting a connection between CMV-induced SA accumulation and changes in tomato–aphid interactions. To determine if any mechanistic relationship exists between SA and CMV-induced resistance to aphid settlement we used *NahG*-transgenic tomato plants, which are depleted in SA [43]. Aphids (*Myzus persicae* or *Macrosiphum euphorbiae*) presented with mock-inoculated plants or plants infected 7 days previously with Fny-CMV settled preferentially on mock-inoculated plants, regardless of whether the infected plants used in the experiment were non-transgenic or were *NahG*-transgenic plants. Interestingly, when aphids of *Myzus persicae*, but not *Macrosiphum euphorbiae*, were presented with *NahG*-transgenic plants infected with Fny-CMV and non-transgenic plants infected with the virus, they settled preferentially on the infected *NahG*-transgenic plants in two out of three experiments. Although this possibly suggests a subsidiary role for SA in influencing interactions between infected plants and *Myzus persicae*, we conclude that the induction of repellence to aphid settlement by Fny-CMV in tomato cannot be critically dependent upon SA.

By later stages of infection Fny-CMV induced wilting, followed by systemic necrosis, and death in *NahG*-transgenic tomato. Similar lethal effects have been observed with *NahG*-transgenic Moneymaker tomato plants infected with either tomato spotted wilt virus or citrus exocortis viroid [60]. Both agents trigger increased SA accumulation in non-transgenic plants [60], and CMV is well known to induce SA biosynthesis and upregulate SA-dependent gene expression in a wide range of susceptible host plants [58,59,70,71,72]. Such results may seem paradoxical since application of exogenous SA to plants, or induction of endogenous SA production prior to inoculation, inhibits infection by CMV [73,74]. Our results with CMV, and those of López-Gresa and colleagues [60] with tomato spotted wilt virus and citrus exocortis viroid, lend support to the idea that inducing production of SA, a resistance-inducing compound, may serve a pro-viral or pro-viroid function by preventing host death, which would be deleterious to these biotrophic infectious agents [75].

## 5. Conclusions

CMV can exert a form of extended phenotype that has been described as ‘attract-and-deter’ [18]. A well-documented example of an ‘attract-and-deter’ phenotype was observed on squash plants infected by Fny-CMV. Infected squash plants emit aphid-attracting VOCs but accumulate aphid-repellent metabolites in their tissues: effects likely to enhance virus acquisition by aphids and encourage their migration to neighbouring plants [18,32]. The phenomenon differs in tomato in that during the period when infected plants are asymptomatic, they attract *Myzus persicae* and are susceptible to settlement but become repellent when symptoms appear, i.e., attraction and deterrence occur over a longer timescale than for squash. Aphids of the solanaceous specialist species *Macrosiphum euphorbiae* are neither consistently attracted nor repelled by pre-symptomatic tomato plants infected with Fny-CMV, but they are repelled by plants later in infection. Although this is a ‘softer’ version of the attract-and-deter phenomenon, it is still likely to promote localised transmission of the virus [18]. The Subgroup II strain LS-CMV did not affect aphid–plant interactions but exchange of genetic material with Fny-CMV allowed the identification of the multifunctional CMV 2b as the major determinant of resistance settling by *Myzus persicae* and the 2a protein as the main determinant for *Macrosiphum euphorbiae* in infected plants. Our work indicated that aphids are responding to a complex set of cues that may inter alia include olfactory and visual stimuli. The 2b protein does not induce effects on aphid–plant interactions via its effects on SA-mediated defensive signalling. Future work will be needed to elucidate the virus-induced changes in defence and metabolism and the precise combination of plant cues that modify the responses of generalist and specialist aphids towards CMV-infected tomato plants.

## Data Availability

The data presented in this study are available in the article and Supplementary Material.

## Supplementary Materials

The following supporting information can be downloaded at: https://www.mdpi.com/article/10.3390/v14081703/s1, Figure S1: The typical appearance of tomato plants following mock-inoculation (Mock) or inoculation with the Fny strain of cucumber mosaic virus (CMV) strain Fny on cotyledons; Figure S2: Mock-inoculated tomato plant data for Figure 1 carried out to control for any additional stimuli or biases in the experimental set-up; Figure S3: Free-choice settling assays indicated that LS-CMV does not induce resistance to aphid settlement in tomato; Figure S4: Aphid free choice tests to determine if signalling dependent upon salicylic acid influences cucumber mosaic virus-induced changes in aphid–tomato interactions; Figure S5: The typical appearances of non-transformed or *NahG*-transgenic tomato plants; Spreadsheet S1: Aphid Data.
