# Supplementary material for: The Effects of Cucumber Mosaic Virus and Its 2a and 2b Proteins on Interactions of Tomato Plants with the Aphid Vectors Myzus persicae and Macrosiphum euphorbiae
Source: Viruses. 2022 Aug 1;14(8):1703. doi: 10.3390/v14081703 (PMC9416248; doi:10.3390/v14081703)
Supplement: Supplementary file 1 [file viruses-14-01703-s001.zip › Revised Supp figures draft VIRUSES .pdf]

Supplementary Figures

The effects of cucumber mosaic virus and its 2a and 2b proteins on interactions of tomato plants with the aphid vectors *Myzus persicae* and *Macrosiphum euphorbiae*

Warren Arinaitwe [1,2], Alex Guyon [1,3], Trisna D. Tungadi [1,4], Nik J. Cunniffe [1], Sun-Ju Rhee [1], Amjad Khalaf [1,5], Netsai M. Mhlanga [1,6], Adrienne E. Pate [1], Alex M. Murphy [1], and John P. Carr [1]*

[1] Department of Plant Sciences, University of Cambridge, Downing Street, Cambridge CB2 3EA, United Kingdom; njc1001@cam.ac.uk, sjrhee825@gmail.com, ap411@cam.ac.uk, amm1013@cam.ac.uk , jpc1005@cam.ac.uk

[2] Current Address; Alliance for Biodiversity-International Centre for Tropical Agriculture (CIAT), Dong Dok, Ban Nongviengkham, Vientiane, Lao Peoples Democratic Republic; W.Arinaitwe@cgiar.org

[3] Current address; Sainsbury Laboratory Cambridge University, Bateman St, Cambridge CB2 1LR, United Kingdom; alex.guyon@slcu.cam.ac.uk

[4] Current address; School of Life Sciences, Keele University, Newcastle ST5 5BG, United Kingdom; trisnat@gmail.com

[5] Current address; Wellcome Sanger Institute, Wellcome Trust Genome Campus, Hinxton, Saffron Walden CB10 1RQ, United Kingdom; ak37@sanger.ac.uk

[6] Current address; National Institute for Agricultural Botany-East Malling (NIAB-EMR), West Malling ME19 6BJ, United Kingdom; Netsai.Mhlanga@niab.com

* Correspondence: jpc1005@cam.ac.uk

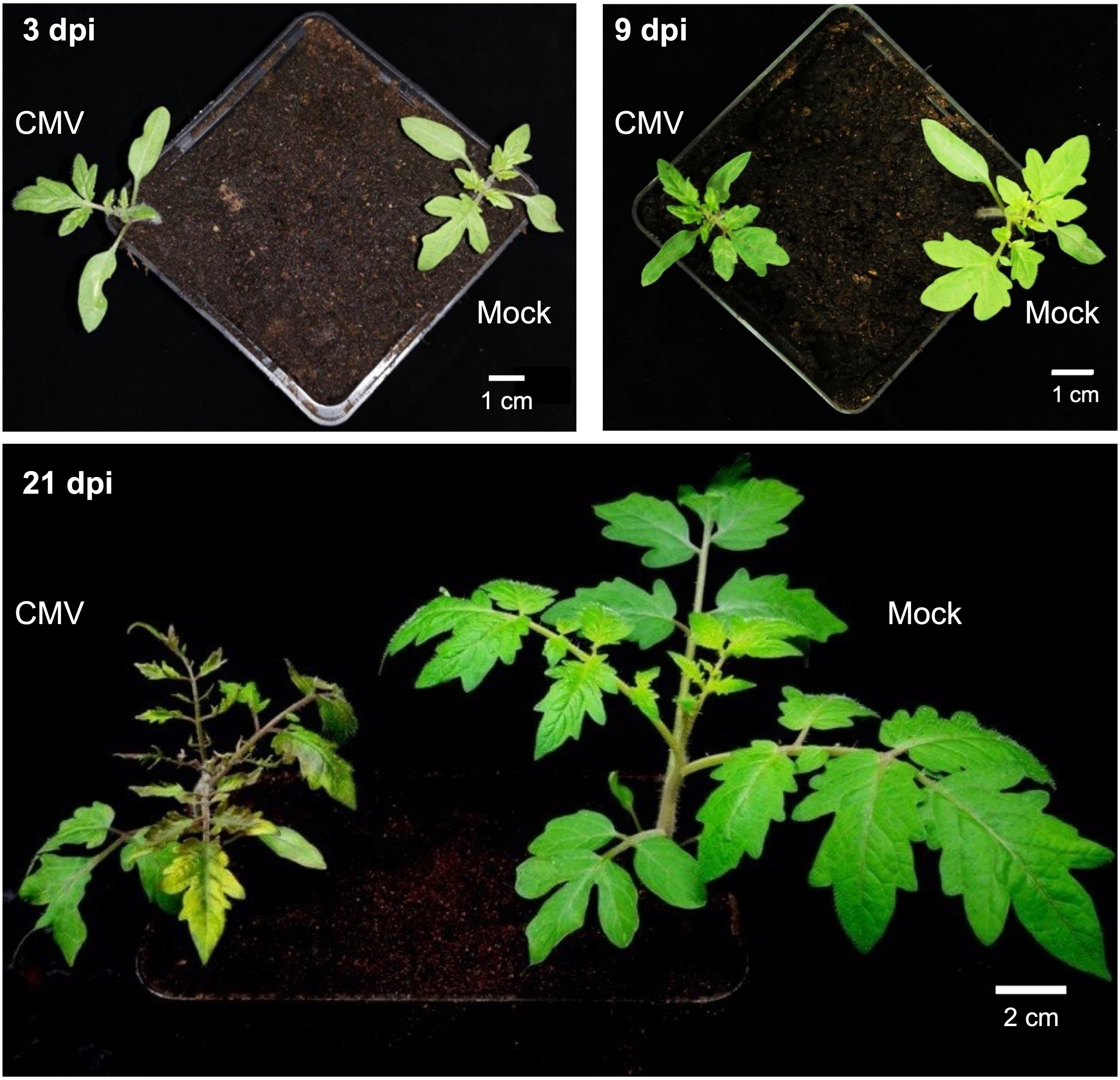

Figure S1. The typical appearance of tomato plants following mock-inoculation (Mock) or inoculation with the Fny strain of cucumber mosaic virus (CMV) strain Fny on cotyledons. Plants were inoculated or mock-inoculated on the cotyledons and photographed at 3, 9 or 21 days post-inoculation (dpi). Systemic infection with CMV was confirmed by ELISA using tissues from non-inoculated true leaves.

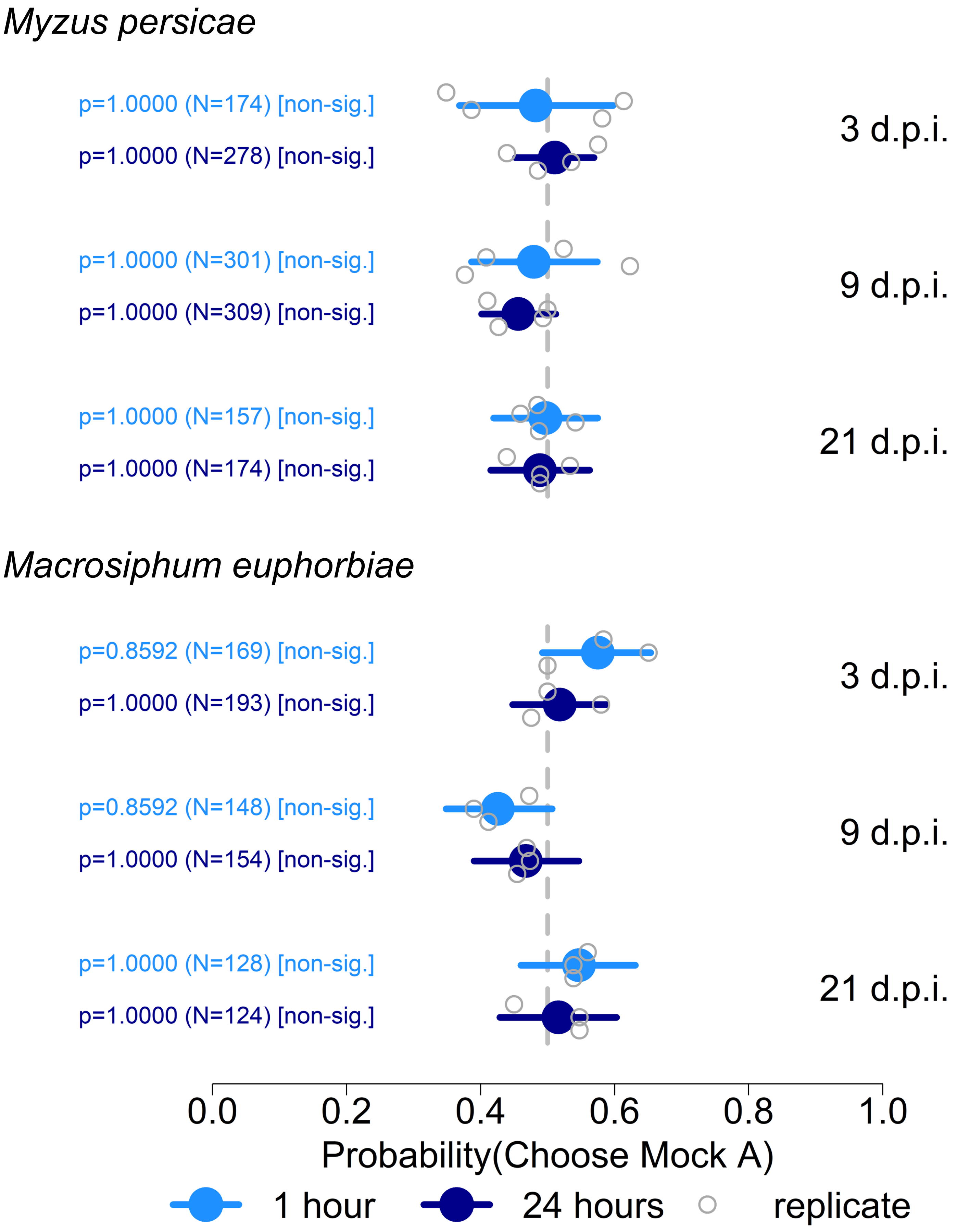

Figure S2 Mock-inoculated tomato plant data for Figure 1 carried out to control for any additional stimuli or biases in the experimental set-up. In these experiments aphids were presented with two mock-inoculated plants (designated plant A and plant B). The x-axis shows the range of the 95% credible interval of the probability of settlement of aphids (*Myzus persicae* or *Macrosiphum euphorbiae*) on infected plants at 1 and 24 h after aphid release, with values for each independent experiment (replicate). The total number of aphids (N) investigated for each comparison is indicated and statistical significance or non-significance determined using binomial tests. Experiments were done at 3, 9, and 21 days following mock inoculation (in parallel with genuinely infected plants: data in Figure 1). The vertical grey dotted lines indicate a probability of 0.5 of settlement on infected versus mock-inoculated plants, i.e., no settlement preference.

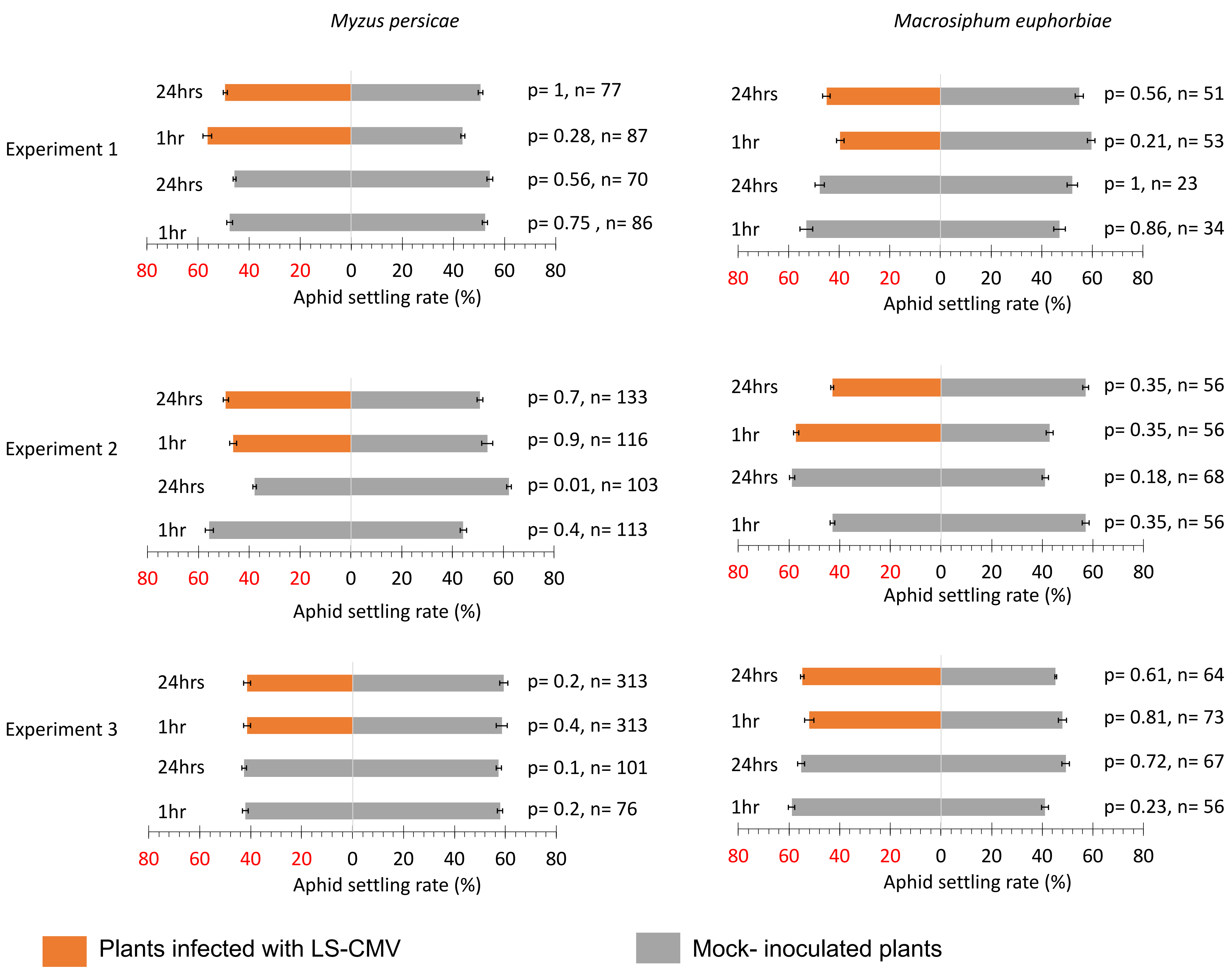

Figure S3 Free-choice settling assays indicated that LS-CMV does not induce resistance to aphid settlement in tomato. In three independent experiments with *Myzus persicae* and *Macrosiphum euphorbiae*, aphids were allowed to settle on either mock-inoculated plants or plants infected with LS-CMV (at 21 days post-inoculation). Aphid settlement was counted at 1 and 24 h post-release. The error bars represent SEM and binomial tests indicated no statistical significance (at $\alpha = 0.05$) in settlement, and n represents the total number of aphids settled per pairwise choice.

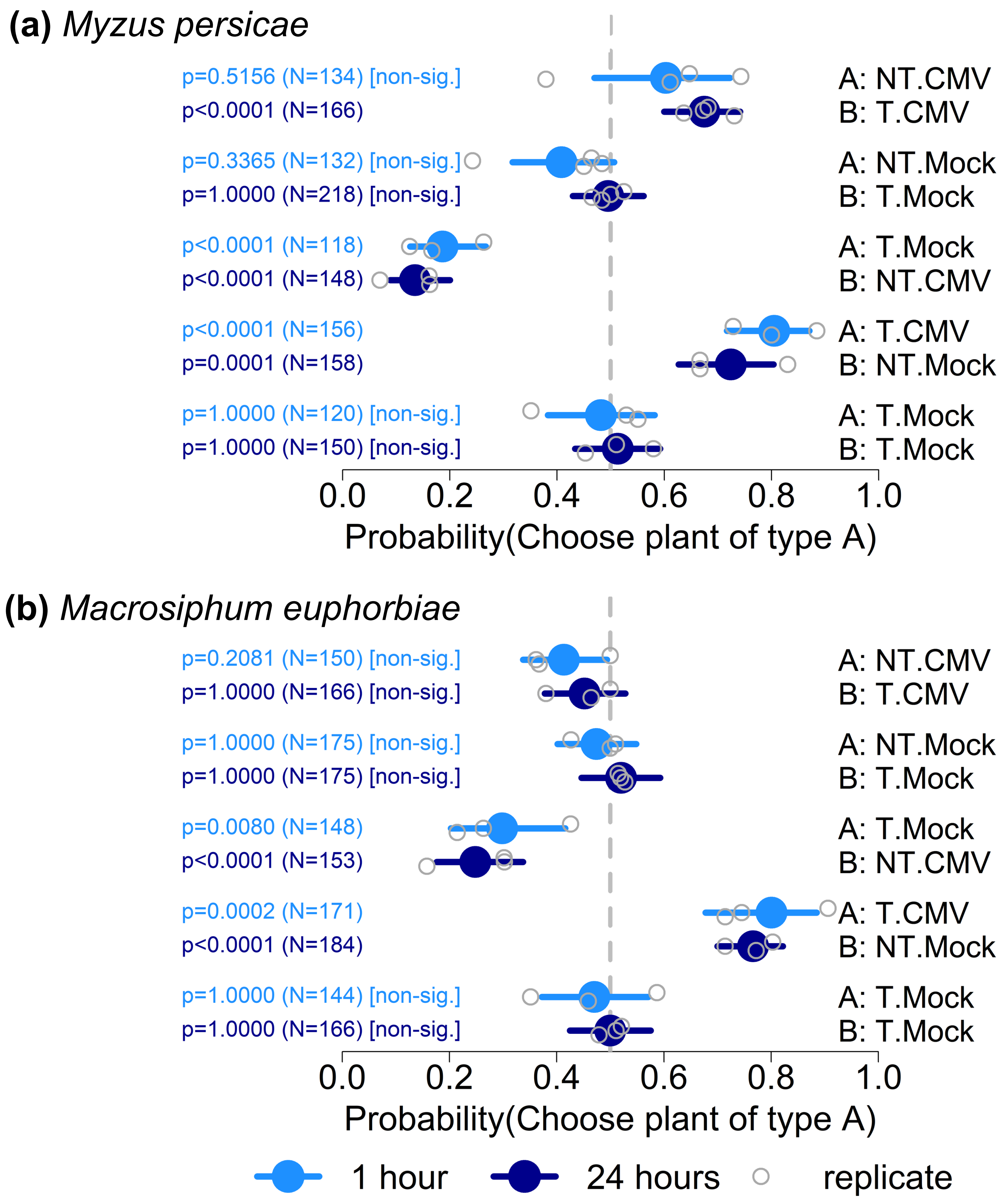

Figure S4 Aphid free choice tests to determine if signalling dependent upon salicylic acid influences cucumber mosaic virus-induced changes in aphid-tomato interactions. Summaries of assays for settlement of **A**. *Myzus persicae* and **B**. *Macrosiphum euphorbiae* on either *NahG*-transgenic (T: unable to accumulate salicylic acid) and non-transgenic (NT) tomato plants that had been mock-inoculated (Mock) or inoculated with Fny-CMV (CMV) 7 days previously (plant A or plant B in various pairwise plant treatment combinations as indicated). Four independent experiments were performed using *Myzus persicae* **(A)** and three with *Macrosiphum euphorbiae* **(B).** The x-axis shows the range of the 95% credible interval of the probability of aphid settlement at 1 and 24 h after aphid release, with values for each independent experiment (replicate). The total number of aphids (N) investigated for each comparison is indicated and statistical significance or non-significance determined using binomial tests. The vertical grey dotted line indicates a probability of 0.5 of settlement on infected versus mock-inoculated plants, i.e., no preference.

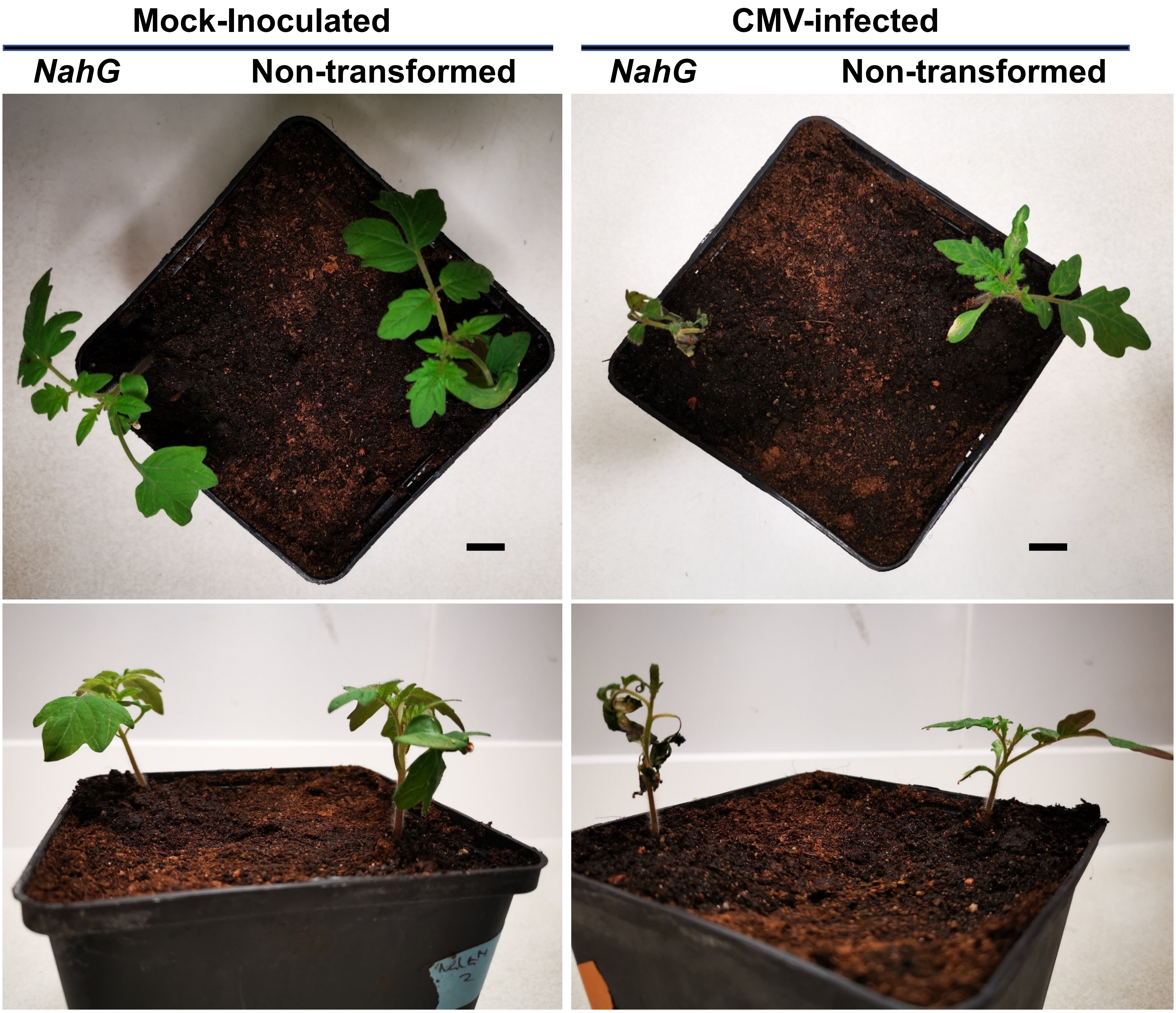

Figure S5. The typical appearances of non-transformed or *NahG*-transgenic tomato plants. Plants were photographed at 10 days following mock-inoculation or inoculation with cucumber mosaic virus (CMV) strain Fny. Scale bars in upper figures indicate 1 cm. The same plants were photographed for top-down and side view photographs.
